# Aptamer-mediated modulation of PAI-1 function reveals a novel mechanism for restoring fibrinolytic balance

**DOI:** 10.1093/narmme/ugag026

**Published:** 2026-05-20

**Authors:** Alicia Matthews, Adi Breitman, Yolanda M Fortenberry

**Affiliations:** Biology Department Case Western Reserve University, Cleveland, OH 44106, United States; Biology Department Case Western Reserve University, Cleveland, OH 44106, United States; Biology Department Case Western Reserve University, Cleveland, OH 44106, United States; Division of Pediatric Hematology, Johns Hopkins University School of Medicine, Baltimore, MD 21287, United States

## Abstract

Plasminogen activator inhibitor-1 (PAI-1) is the principal regulator of fibrinolysis, acting by inhibiting tissue-type and urokinase-type plasminogen activators (tPA/uPA). Dysregulated PAI-1 activity contributes to diverse pathologies, including cancer, cardiovascular disease, and fibrosis, underscoring its therapeutic potential. We previously identified R10-4, an RNA aptamer that attenuates PAI-1’s antiproteolytic activity against tPA by destabilizing covalent complex formation. To elucidate its inhibitory mechanism, we combined plasminogen activation assays with *in silico* modeling to map residue-level interactions. Systematic truncation of R10-4 revealed two regions critical for inhibition: (i) the reactive center loop, encompassing the s5A strand, and (ii) the vitronectin-binding domain. Removal of 17 nucleotides from the 3′ end disrupted aptamer engagement, significantly altering R10-4′s inhibitory activity. These findings define the structural basis for R10-4 modulation of PAI-1 function, revealing a novel aptamer-mediated mechanism of inhibition. By targeting key functional domains, R10-4 emerges as a promising prototype therapeutic for correcting fibrinolytic dysregulation in thrombotic and fibrotic disease.

## Graphical Abstract

**Graphical Abstract:**
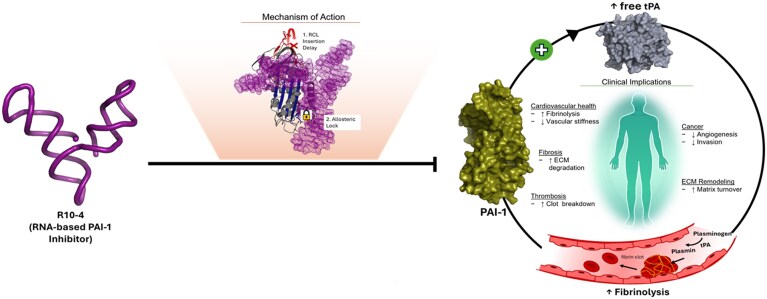

## Introduction

Plasminogen activator inhibitor-1 (PAI-1) is the primary physiological inhibitor of tissue-type and urokinase-type plasminogen activators (tPA and uPA), thereby serving as a central regulator of fibrinolysis. By blocking plasminogen activation, PAI-1 prevents fibrin degradation and maintains hemostatic balance [1]. Elevated PAI-1 expression and activity have been strongly associated with pathological processes, including thrombosis, cardiovascular disease, fibrosis, and multiple cancers, where dysregulated fibrinolysis contributes to aberrant clot stability, extracellular matrix remodeling, and tumor progression [1–6]. Given its broad pathological involvement, PAI-1 has emerged as an attractive therapeutic target.

**Figure 1.**
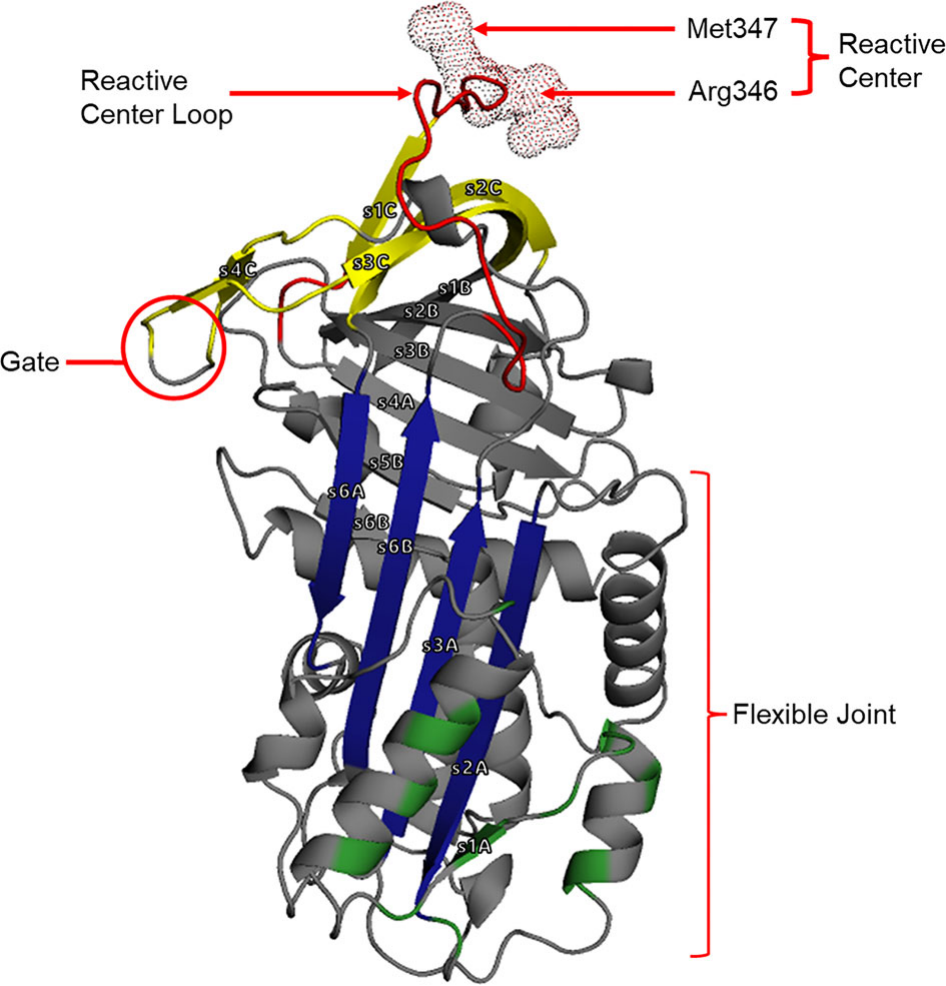
Cartoon representation of active PAI-1 (PDB ID: 1B3K). Structural regions implicated in conformational transitions that interact with PAI-1 targeting aptamers, including the flexible joint and gate, are annotated with red dotted circles. The RCL is shown in red, with the reactive center residues Met347 and Arg346 highlighted as red spheres. β-sheets A and C are color-coded in blue and yellow, respectively. Vitronectin-binding sites are indicated in green and localized within the flexible joint region. Structural visualization and annotation were performed via PyMOL.

Structurally, PAI-1 consists of three β-sheets (A–C, denoted as s[#]A, s[#]B, and s[#]C), nine α-helices, and a reactive center loop (RCL) (Fig. 1). This structural framework includes several key domains that regulate its conformational changes, including the flexible joint, breach, hinge, gate, and shutter region [2]. The flexible joint region comprises the vitronectin-binding domain (VNBD), which is crucial for PAI-1’s stability. Here, we present an RNA aptamer that inhibits PAI-1 activity by targeting several structural regions, including the RCL, gate, flexible joint region, VNBD, and β-sheets A and C (Fig. 1).

RNA aptamers are short single-stranded oligonucleotides that fold into unique, tertiary structures around a specific target (i.e. protein). Aptamers are frequently likened to antibodies due to their comparable or enhanced target affinity and specificity [7]. They also offer advantages, including ease of synthesis, chemical modifiability, and low immunogenicity. Aptamers are generally synthesized using a technique referred to as systematic evolution of ligands by exponential enrichment (SELEX) [7]. Several PAI-1-specific RNA aptamers have been identified that interfere with its inhibitory function on plasminogen activators, thereby promoting fibrinolysis and suppressing PAI-1-mediated effects on cell migration, invasion, and angiogenesis [8–11]. In particular, the PAI-1-specific RNA aptamer R10-4 blocks PAI-1’s interaction with tPA, effectively restoring fibrinolytic activity [12]. However, the mechanism by which R10-4 exerts its inhibitory activity remains unclear and is addressed in the current study.

**Figure 2.**
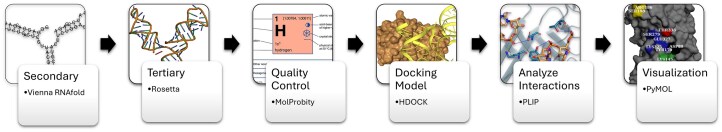
Computational workflow for predicting the three-dimensional structure and docking interactions of the RNA aptamer R10-4 and its truncations with active PAI-1 (PDB ID: IB3K). The pipeline integrates freely available web-based tools across six stages: secondary structure prediction (ViennaRNA), tertiary structure modeling (Rosetta), hydrogen atom addition and geometry validation (MolProbity), protein–RNA docking (HDOCK), interaction profiling (PLIP), and structural visualization (PyMOL).

The integration of molecular simulation methodologies has emerged as a powerful strategy for informing and refining aptamer design. A critical aspect of this process is elucidating how the unique tertiary structures of aptamers engage with their targets to modulate activity. Computational approaches, such as *in silico* strategies, have become pivotal for characterizing these interactions. *In silico* methods are freely available bioinformatic simulation workflows that enable the identification of structural motifs and interaction profiles within ligand-receptor complexes, such as aptamer-protein assemblies. Prior studies have demonstrated that *in silico* approaches can enhance aptamer screening and validation by incorporating preselection procedures that reduce the initial SELEX library, as well as by guiding chemical modifications to optimize molecular recognition and functional efficacy [13–15]. In the present study, we employed an *in silico* modeling workflow to predict the secondary and tertiary folding of PAI-1-targeting aptamers and to generate a comprehensive interaction profile of the aptamer: PAI-1 complex (Fig. 2).

Building upon these insights, we conducted a comprehensive structure–function analysis of truncated derivatives of R10-4. By integrating biochemical characterization with molecular simulation, we assessed the functional consequences of R10-4 on PAI-1–mediated cellular processes. Together, these integrated approaches enabled us to define the structural determinants required for the antiproteolytic activity of R10-4 and to visualize aptamer–PAI-1 interactions. We elucidate a bivalent binding mechanism in which R10-4 binds to two critical regions of PAI-1, the RCL/strand 5A and the VNBD, thereby inhibiting its activity. These findings deepen our mechanistic understanding of how R10-4 achieves potent inhibition by targeting two distinct functional domains of PAI-1.

## Materials and methods

### Reagents

The human stable PAI-1 mutant containing the mutations K154T, Q319L, M354I, and N150H was purchased from Innovative Research, Inc. These mutations “lock” PAI-1 in an active state and delay the spontaneous conversion of PAI-1 to its latent conformation. As demonstrated previously, the aptamers bind equally well to both wild-type PAI-1 and the stable PAI-1 mutant (Demare *et al*., 2014). The chromogenic substrates, S2288^TM^ and S2251^TM^, were purchased from DiaPharma. Plasminogen activator, tPA, human plasminogen, and PAI-1 monoclonal antibody were purchased from Innovative Research, Inc.

### R10-4 and truncated variants synthesis

R10-4 was developed using a previously described protocol, SELEX [10]. R10-4 truncated variants were generated by designing primers that excluded specific residues, reducing the full-length R10-4 from the 3′ end. The following 3′ primers were used for their respective variant: T2 – CGAGTCGTCTACTCGA + TCGAGTAGACGACTCG, T3 – GTCTACTCGACGCCTA + TAGGCGTCGAGTAGAC, T4 – CGACGCCTACGTTAT + ATAACGTAGGCGTCG, T5 – CTACGTTATGCAGGC + GCCTGCATAACGTAG. Complementary DNAs (cDNAs) were transcribed to RNA using DuraScribe T7 transcription kit (LGC Biosearch Technologies) according to the manufacturer’s protocol. Briefly, 1 μg of cDNA was incubated with 1 μl each of GTP, ATP, UTP, CTP (the pyrimidines were modified with a 2′-fluoro group), and DTT in transcription buffer. The reaction was incubated overnight at 37°C, after which, DNase I (1 μl) was added to remove the DNA template. Transcripts were extracted using phenol-chloroform/isoamyl alcohol, and an equal volume of 2× formamide loading buffer was added, followed by incubation at 65°C for 5 min. The RNA was then cleaned and concentrated using the Zymo RNA Concentrator kit as per the manufacturer’s protocol. The purity and integrity of the RNA product were assessed by gel electrophoresis, and the concentration was determined using a Nanodrop (Thermo Fisher Scientific, Waltham, MA).

### Surface plasmon resonance

Surface plasmon resonance (SPR) experiments were performed using a Reichert SPR to evaluate the interaction between PAI-1 and the R10-4 aptamers. The experiments were conducted at 25°C using a CM5 sensor chip in SPR buffer (10 mM HEPES, 150 mM NaCl, 3 mM ethylenediaminetetraacetic acid, 0.05% Tween-20, pH 7.4). All buffers were filtered through a 0.22 µm membrane and degassed before use. Ligand immobilization: PAI-1 was immobilized on the sensor chip surface via amine coupling. Briefly, the chip was activated with a 1:1 mixture of 0.2 M N-hydroxysuccinimide and 0.2 M N-ethyl-N' (3-dimethylaminopropyl) carbodiimide at a flow rate of 25 µl/min for 7 min. PAI-1 was diluted to 50 µg/ml in immobilization buffer (10 mM sodium acetate, pH 4.5) and injected onto the activated surface until a desired immobilization level was achieved. Residual reactive groups were blocked with 1 M ethanolamine (pH 8.5) for 7 min. Binding: A concentration series of R10-4 ranging from 1 to 5 nM was prepared in running buffer. Analyte solutions were injected at a flow rate of 25 µl/min for 100 s to monitor association, followed by a dissociation phase of 300 s. A blank injection of running buffer alone was included as a reference to correct for nonspecific signals. The sensor chip was regenerated between binding cycles using a regeneration buffer containing NaOH for 30 s to remove residual bound analyte. Data analysis: Sensorgrams were analyzed using TraceDrawer to determine kinetic and equilibrium binding parameters. Data are fit to a 1:1 Langmuir binding model to calculate the association rate constant (*k*_a_), dissociation rate constant (*k*_d_), and equilibrium dissociation constant (*K*_D_). The reference channel and blank injections were used to subtract nonspecific binding and buffer-related artifacts. This method enabled the precise characterization of the interaction between PAI-1 and the aptamer R10-4, providing insights into binding affinity and interaction kinetics.

### *In silico* predictive modeling

To identify functional motifs within the R10-4 aptamer that mediate PAI-1 inhibition, we employed a multi-step *in silico* workflow using freely available bioinformatic tools. This approach enabled comparative analysis of R10-4 and its truncated variants, allowing us to correlate regional sequence loss with reduced activity and pinpoint key interaction sites. Aptamer sequences were first submitted to the ViennaRNA Webserver (http://rna.tbi.univie.ac.at/cgi-bin/RNAWebSuite/RNAfold.cgi) using default parameters to predict secondary structure in dot-bracket notation. These secondary structure predictions were then used as input for Rosetta FARFAR2 (https://rosie.graylab.jhu.edu/farfar2) to generate three-dimensional models of R10-4 and its truncated variants. The lowest-energy structure was selected for further refinement. Hydrogen atoms were added using MolProbity (http://molprobity.biochem.duke.edu/), incorporating Asn/Gln/His side-chain flips and optimizing X–H bond lengths based on electron cloud geometry. All-atom contacts and stereochemical parameters were evaluated to ensure structural integrity.

The PDB files of the hydrogen-complete aptamer models were then submitted to the HDOCK server (http://hdock.phys.hust.edu.cn) for protein–RNA docking. Active PAI-1 was retrieved from the RCSB Protein Data Bank (PDB ID: 1B3K) and designated as the receptor. R10-4 was designated as the ligand. Docking outputs were ranked by energy score, and the lowest-energy complex was selected for downstream analysis.

Protein–RNA interactions were characterized using the protein–ligand interaction profiler (PLIP) (https://plip-tool.biotec.tu-dresden.de/plip-web/plip), which identified noncovalent contacts including hydrogen bonds, π-stacking, and salt bridges. Final structural visualizations and residue-level annotations were performed locally using PyMOL. *In silico* workflow is shown in Fig. 2.

### Direct tPA activity assay

All activity assays were performed in 96-well bovine serum albumin-coated plates. For the direct tPA activity assay, PAI-1 (40 nM) was preincubated with the RNA aptamer (50 nM) in HNPN buffer (20 mM HEPES, 150 mM NaCl, 0.01%, polyethylene glycol (PEG), and 0.0055% sodium azide) supplemented with 2.5 mM CaCl_2_ at room temperature for 10 min. Recombinant tPA (5 nM) was then added, and the reaction mixture was incubated at 37°C for an additional 10 min. Residual tPA activity was quantified by measuring the cleavage of the chromogenic substrate S-2251^TM^ (DiaPharma). Immediately following substrate addition, absorbance at 405 nm was monitored using a VersaMax microplate reader (Molecular Devices). Kinetic measurements were recorded every 15 s for 20 min. For all activity assays, prior to the reaction, the RNA aptamers were heated at 70°C for 5 min, followed by incubation on ice.

### Plasminogen activation assay

Plasmin generation was measured to assess the effect of RNA aptamer–mediated inhibition of PAI-1 on tPA-dependent plasminogen activation. Human PAI-1 (40 nM) was preincubated with RNA aptamer inhibitor (50 nM) in HNPN buffer supplemented with 2.5 mM CaCl_2_ at room temperature for 10 min to allow complex formation. Following incubation, recombinant tPA (5 nM) and human Glu-plasminogen (0.5–1 µM) were added to initiate the reaction. The mixture was incubated at 37°C, and plasmin generation was monitored by cleavage of the chromogenic substrate S-2288^TM^ (DiaPharma), which is specific for plasmin. Immediately after substrate addition, absorbance at 405 nm was recorded using a VersaMax microplate reader. Kinetic measurements were acquired every 15 s for 30–60 min. The rate of plasmin generation was calculated from the linear portion of the absorbance curve. Control reactions included tPA and plasminogen in the absence of PAI-1, as well as PAI-1 without aptamer, to determine baseline and maximal inhibition, respectively.

### PAI-1–tPA complex formation assay

Covalent complex formation between PAI-1 and tPA was evaluated by sodium dodecyl sulfate–polyacrylamide gel electrophoresis (SDS–PAGE) analysis and immunoblot analysis. Briefly, human PAI-1 (400 nM) was incubated with recombinant tissue-type plasminogen activator (tPA; 500 nM) in HNPN buffer (20 mM HEPES, 150 mM NaCl, 0.01% PEG, and 0.0055% sodium azide) supplemented with 2.5 mM CaCl_2_. Where indicated, RNA aptamers (0–500 nM) were incubated with PAI-1 at room temperature for 10 min prior to the addition of tPA. The aptamer clones (50–500 nM) were incubated with PAI-1 (500 nM) for 10 min at room temperature. Subsequently, tPA (400 nM) was added to the reaction and then incubated at 37°C for 5 min. Following the addition of tPA, reactions were incubated at 37°C for 10–15 min to allow complex formation. The reaction was stopped by adding Laemmli sample buffer containing β-mercaptoethanol and boiled for 5 min. The samples were then subjected to SDS–PAGE analysis or western blotting using a monoclonal PAI-1 antibody. Reactions were then added to a non-reducing SDS sample buffer and immediately heated at 95°C for 5 min. Samples were resolved on 8%–10% SDS–PAGE gels under non-reducing conditions. Proteins were visualized by Coomassie Brilliant Blue staining. The formation of the SDS-stable PAI-1–tPA complex was identified as a high-molecular-weight band (∼110 kDa), distinct from free PAI-1 (∼45 kDa) and tPA (∼70 kDa). Densitometric analysis was performed using ImageJ software to quantify complex formation relative to total PAI-1. Control reactions included PAI-1 or tPA alone, as well as reactions lacking aptamer, to assess baseline complex formation.

### Statistical analysis

Data are presented as mean values with standard deviation. Significance among the groups relative to “no aptamer” control groups was tested using an unpaired Student’s *t*-test. The test was calculated using GraphPad Prism software (*P*-values < .05 were considered statistically significant).

## Results and discussion

### R10-4 truncation produces smaller aptamer variants with reduced structural complexity

**Figure 3.**
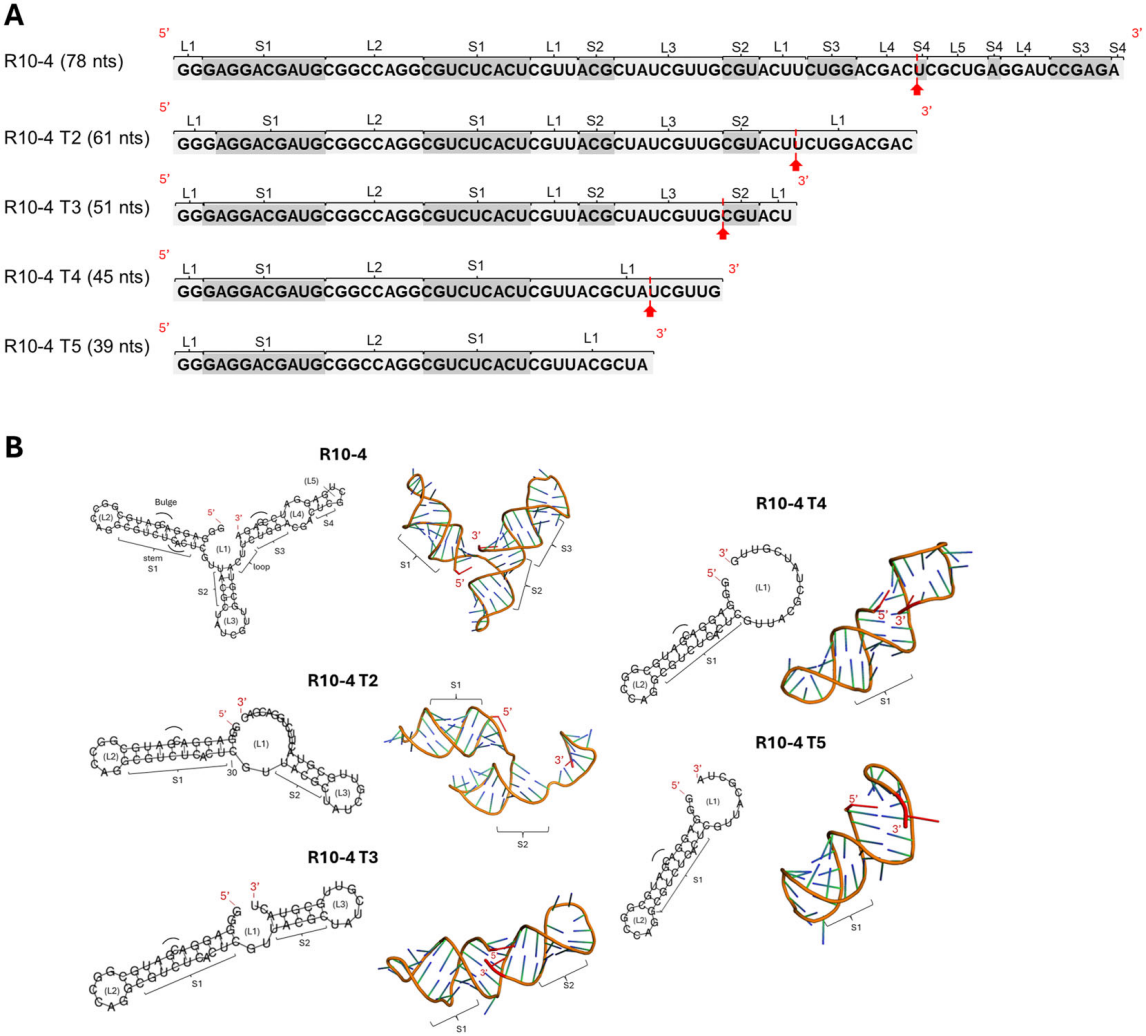
Sequence alignment and predicted secondary and tertiary structures of R10-4 and its truncated variants (T2–T5). (**A**) Aligned nucleotide sequences of the full-length R10-4 aptamer and its truncated derivatives (T2–T5), with the length of each construct indicated. Red arrows and dotted lines mark the regions removed to generate each successive truncation. (**B**) Predicted secondary and tertiary structures of R10-4 and its variants. Left panels show secondary structure models generated using the ViennaRNAfold Web server. Structural elements are annotated from 5′ to 3′ direction, beginning with the central loop (L1) and first stem (S1), and progressing through loops L2–L5 and stems S2–S4. Bulges are indicated by semi-circles. Right panels display the corresponding tertiary structures predicted using Rosetta FARFAR2 and visualized in PyMOL. The 5′ and 3′ termini of each RNA are highlighted in red.

Aptamers are relatively large and structurally complex, which can reduce stability, increase synthesis costs, and hinder translational potential [14, 16]. Systematic truncation is a widely used strategy to identify the minimal functional domains required for aptamer activity [14, 16]. Truncated aptamers often retain, or in some cases improve, binding affinity and inhibitory function if the essential structural motifs are preserved, while offering advantages such as improved manufacturability and more favorable pharmacological properties [14, 16]. Based on this rationale, we aimed to define the core functional domain of R10-4 by systematically truncating the sequence at the 3′ end Table 1; Fig. 3A).

**Table 1.** RNA sequences and binding affinities of full-length R10-4 and truncated variants (T2–T5)

Aptamer	RNA sequence	*K*_d_ (nM)
R10-4	*gggaggacgaugcgg*ccaggcgucucacucguuacgcuaucg uugcguacuucug*gacgacucgcugaggauccgaga*..(((((.(((((......))))))).)))..(((((.......)))))..((((((...(((...)))..))).)))	48.5+3.2
R10-4 T2	*gggaggacgaugcgg*ccaggcgucucacucguuacgcu aucguugcguacuucuggacgac -17..(((((.(((((......))))))).)))..(((((.......)))))............	43.1+4.0
R10-4 T3	*gggaggacgaugcggc*caggcgucucacucguu acgcuaucguugcguacu -27..(((((.(((((......))))))).)))..(((((.......)))))..	73.9+5.7
R10-4 T4	*gggaggacgaugcgg*ccaggcgucucacu cguuacgcuaucguug -33..(((((.(((((......))))))).)))...............	65.5+4.1
R10-4 T5	*gggaggacgaugcgg*ccaggcgucu cacucguuacgcua -39..(((((.(((((......))))))).))).........	361.6+9.8

**R10-4 was systematically truncated from the 3′ end. The binding affinity to PAI-1 of R10-4 and its variants was determined using SPR. The data are the average of at least three independent experiments. The numbers represent the number of nucleotides removed.

Truncation mutants were generated using primers designed to exclude defined regions of the 3′ end, producing progressively shortened forms of the original aptamer (Table 1 and Fig. 3A). Secondary structures were predicted and visualized using ViennaRNA Fold, yielding minimum free-energy configurations that revealed distinct stem–loop architectures [17]. Tertiary structures were modeled with Rosetta FARFAR2, and the lowest-energy conformation was selected for each variant [18–20]. These models enabled the visualization of structural features such as curvature and backbone orientation (Fig. 3B).

The resulting structures showed a preserved core stem region and loop design, with variation in overall topology depending on the truncation length (Fig. 3B). R10-4 forms a three-branched conformation consisting of five loops (L1–L5) and four stems (S1–S4), with a central loop (L1). Loops L1 and L2, as well as stem S1, were retained across all truncation variants. Variants T2 and T3 displayed partial branching, maintaining three loops (L1–L3) and two stems (S1–S2). Notably, the central loop (L1) in T3 was reduced in size, narrowing the angle between stems and producing a more linear conformation compared with T2. In contrast, the T4 and T5 variants adopted even more linear conformation.

### Truncation of R10-4 does not significantly alter binding affinity

We next evaluated the binding affinities of all truncation variants using SPR. Interestingly, no substantial loss of binding affinity was observed until ~39 nucleotides were removed from the 3′ end (variant R10-4T5), indicating that sequences lost in T5 are critical for PAI-1 interaction. SPR analysis showed that R10-4T2, T3, and T4 maintained high-affinity binding comparable to R10-4, suggesting that the essential binding motif is contained within the shared structural region (Table 1, Supplementary Fig. S1, and Fig. 3). These results imply that truncation does not enhance R10-4’s affinity for PAI-1, which already possesses an optimized binding interface.

**Figure 4.**
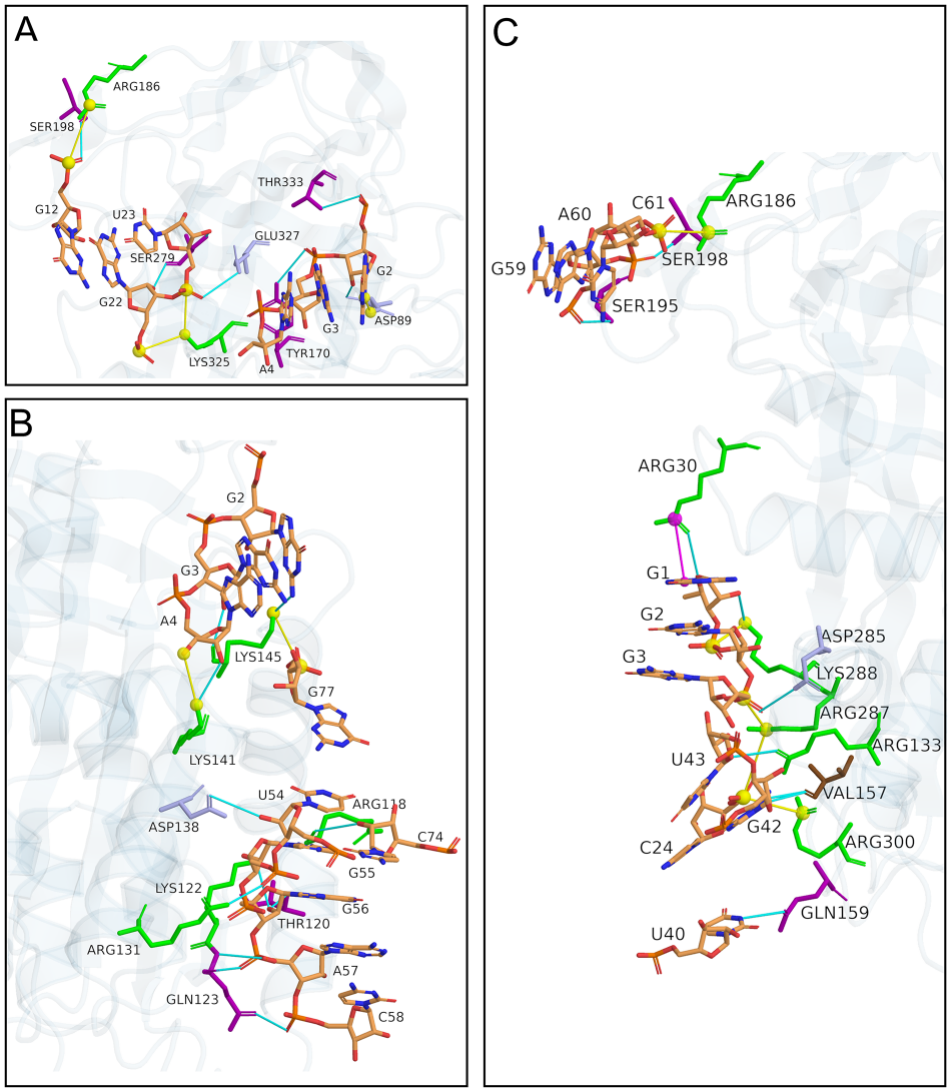
Residue-level interactions between R10-4/T2 and active PAI-1 (PDB ID: 1B3K). (**A**) Interactions between the full-length R10-4 aptamer and PAI-1, highlighting contacts across the primary binding interface. (**B**) R10-4 interactions specifically within the VNBD of PAI-1. (**C**) Interactions by the R10-4 T2 with PAI-1. Across all panels, PAI-1 residues are color-coded by chemical properties: positively charged residues (Lys, Arg, His) in green; negatively charged residues (Asp and Glu) in light blue; polar residues (Gln, Thr, Ser, and Tyr) in purple; and nonpolar residues (Val) in brown. Interaction types are indicated as: cyan lines denote hydrogen bonds, yellow lines denote salt bridges, and purple lines denote π-cation interactions. Spheres mark the calculated charge centers of each interacting residue in salt bridges and π-cation interactions. RNA aptamer atoms are shown in orange, with oxygen and nitrogen atoms highlighted in red and blue, respectively.

We then investigated the molecular interactions between the RNA aptamers and PAI-1 that contributed to the binding affinity. Tertiary structures of R10-4 and its truncated variants were docked with active PAI-1 (PDB ID: 1B3K) using HDOCK, and interaction profiles were analyzed using PLIP, respectively [21–23]. Molecular docking revealed that aptamer-PAI-1 interactions are predominantly polar and electrostatic, driven by negatively charged aptamer residues engaging positively charged, hydrophilic amino acids on PAI-1 (Fig. 4A). R10-4 aptamer made several hydrogen-bonding interactions, with the strongest being with polar amino acids located in the VNBD of PAI-1 (Fig. 4B). Truncations, T3–T5, also made several polar interactions in this region via hydrogen bonding (Supplementary Fig. S2). The salt-bridge interactions involved positively charged amino acids, mainly lysine and arginine, with full-length R10-4 forming six, T2 and T5 forming five, and T3 and T4 forming three (Supplementary Fig. S2). Polar, hydrophilic residues were highly represented among the interacting amino acids. Lysine accounted for 37% and 42% of all interactions in the full-length R10-4 and T4 variants, respectively, while arginine predominated in T2, T3, and T5, contributing 47%, 31%, and 31% of their respective interaction profiles (Supplementary Fig. S2). Lastly, T2 was the only variant to form a pi cation interaction (Supplementary Fig. S2 and Fig. 4C). These findings show that the PAI-1/R10-4 complex is primarily stabilized through polar interactions. These data align with the affinity data, indicating no significant differences in molecular interactions between full-length R10-4 and the truncation variants that would significantly alter binding affinity.

### Both R10-4 and R10-4T2 retain inhibitory activity against PAI-1, while truncation variants R10-4T3–T5 exhibit a marked loss of inhibition

Given that the full-length R10-4 and the truncation variants bind to PAI-1 with similar affinities, we suspected that they would have similar effects on PAI-1’s inhibitory activity. To assess this, we evaluated whether the truncation variants could block PAI-1’s interaction with tPA. Our previous findings demonstrated that full-length R10-4 inhibits PAI-1 from forming a stable, covalent complex with tPA, potentially by shifting PAI-1 toward the substrate pathway [10]. Therefore, we conducted activity assays on full-length R10-4 and its truncated variants (T2–T5) against PAI-1.

**Figure 5.**
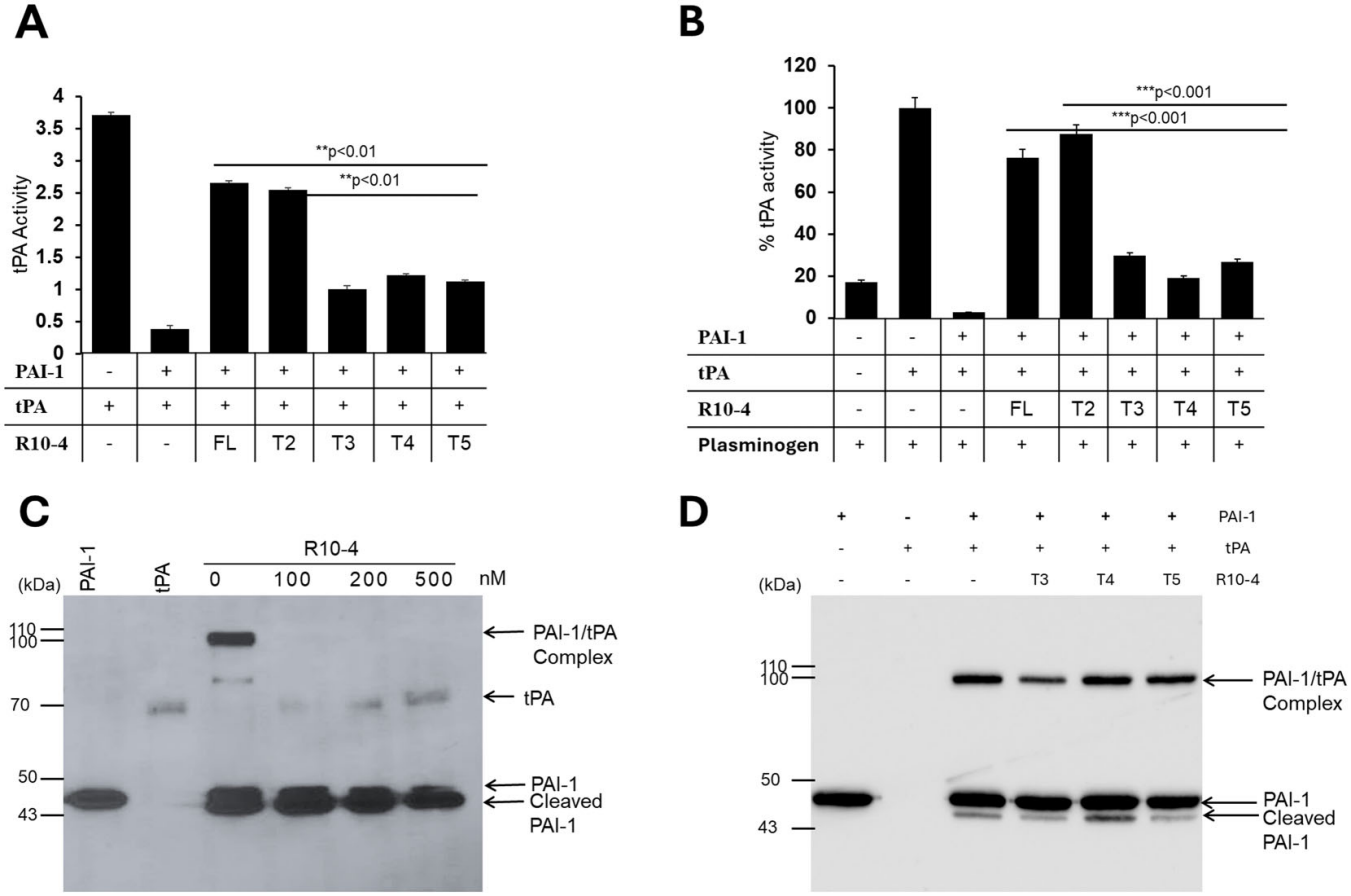
Inhibition of PAI-1 activity by full-length R10-4 (FL) and R10-4 truncation variants. (**A**) Full-length R10-4 (FL) and truncation mutants were evaluated for their ability to inhibit the interaction between PAI-1 and tPA. In the absence of PAI-1 and aptamer, tPA activity is maximal. As expected, the addition of PAI-1 (40 nM) results in a significant reduction in tPA activity. Both R10-4 (FL) and the truncation variant R10-4T2 (50 nM) restore ~80% of tPA activity, whereas truncation variants T3–T5 fail to restore tPA activity to levels comparable to full-length R10-4. (**B**) The tPA-mediated plasmin generation is markedly inhibited in the presence of PAI-1 (40 nM). Consistent with panel A, R10-4 (FL) and R10-4T2 (50 nM) effectively restore plasmin generation, while truncation variants R10-4T3–T5 are significantly less efficient at rescuing plasmin activity. (**C**) SDS–PAGE gel showing the disruption of the PAI-1/tPA complex at increasing concentrations of R10-4 aptamer. (**D**) Immunoblot analysis showing that the truncation variants form a stable PAI-1/tPA complex, similar to the PAI-1/tPA complex in the absence of aptamers, indicating that PAI-1 remains active in the presence of the truncated variants.

PAI-1 activity was assessed using both a direct assay (tPA activity alone) and an indirect assay (tPA-dependent plasminogen activation). As expected, PAI-1 inhibits ~90% of tPA activity in the absence of aptamers (Fig. 5A). Both R10-4 and R10-4 T2 restored tPA activity to ∼75%, demonstrating that each inhibits PAI-1. In contrast, truncation variants R10-4 T3–T5 did not inhibit PAI-1 activity, showing significantly less recovery of tPA activity compared with full-length R10-4. These results indicate that although R10-4T3–T4 retain PAI-1 binding, they are unable to disrupt PAI-1′s interaction with tPA.

To further confirm these findings, we evaluated tPA's ability to convert plasminogen to plasmin. As shown in Fig. 4B, tPA efficiently activates plasminogen, while PAI-1 strongly inhibits this reaction. Consistent with results from the direct assay, R10-4 and R10-4 T2 restored plasminogen activation by inhibiting PAI-1, with R10-4 inhibiting PAI-1 in a concentration-dependent manner, further supporting its functional activity (Fig. 5C). Similar to the direct assays, R10-4 T3–T5 failed to inhibit PAI-1, resulting in reduced plasminogen activation.

We then assessed if the truncation variants disrupt the PAI-1/tPA stable complex. We previously showed that R10-4 disrupts this complex, leading to an increase in cleaved PAI-1 [10]. We repeated this experiment with similar results as shown in Fig. 5C. However, in Fig. 5D, the SDS stable complex between PAI-1 and tPA in the presence of the truncation variants remains intact, indicating that these aptamers do not effectively inhibit PAI-1 from interacting with tPA.

Collectively, these data show that R10-4 and R10-4 T2 effectively disrupt the PAI-1/tPA interaction, allowing tPA to process both artificial (tPA substrate) and physiological (plasminogen) substrates. The truncated variants (T3–T5) may engage in a region that is not critical for antiproteolytic activity or for the PAI-1/tPA interaction, accounting for their apparent increase in binding affinity and lack of inhibitory activity.

### R10-4 and T2 engage critical PAI-1 regions, corroborating their inhibitory activity

Previous studies have shown that the RCL region and some residues in the VNBD are important for the PAI-1/tPA interaction; however, whether R10-4 or the truncation variants bind to these regions is unknown [2, 8–11]. Hence, our next step was to map the region on PAI-1 to which these aptamer variants bind, as this will provide the necessary evidence to assess the mechanism by which R10-4 inhibits PAI-1′s inhibitory activity.

**Figure 6.**
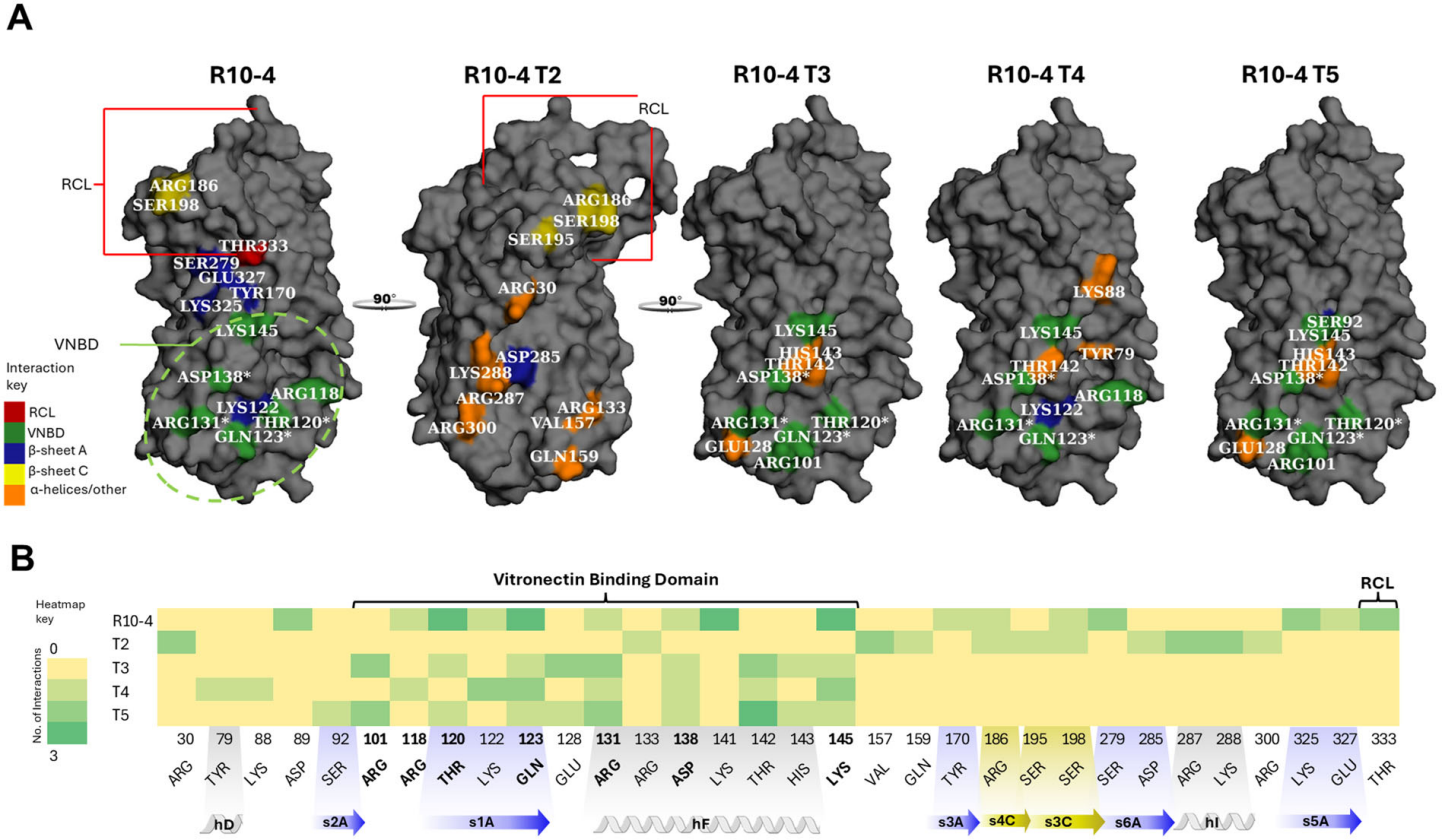
Structural and sequence-level mapping of R10-4 and its derivatives (T2–T5) interacting with the PAI-1 interface. (**A**) Surface representation of active PAI-1 (PDB ID: 1B3K), highlighting residues contacted by each aptamer. Interaction sites are color-coded according to structural and functional regions: the RCL in red, the VNBD in green, β-sheet A in blue, β-sheet C in yellow, and α-helices/non-structured regions in orange (see interaction key). Interacting residues are labeled. Residues marked with an asterisk interact with vitronectin but also reside within a defined structural region: Arg120 and Arg123 are on β-sheet s1A, and Arg131 and Arg138 on helices hF. (**B**) Heatmap showing the number of noncovalent interactions between PAI-1 and each aptamer variant. Dark green denotes up to three contacts per residue; yellow indicates no interaction (heatmap key). The PAI-1 sequence below the heatmap is annotated to indicate structural and functional motifs, with β-sheets A and C shown in blue and yellow, respectively, and α-helices shown in gray. PAI-1 surface visualization was performed in PyMOL.

Therefore, we evaluated whether R10-4 and its truncated variants engage residues with potential regulatory significance by mapping residue-level interactions between active PAI-1 (PDB ID: 1B3K) and each aptamer variant (T2–T5) using predictive modeling analysis. R10-4 exhibited the most extensive interaction footprint, engaging residues across multiple functional domains, including the VNBD, RCL, and several β-sheets (Fig. 5A). R10-4 was the only aptamer to interact with the RCL, specifically Thr333. Within the β-sheets, R10-4 formed five contacts, including β-sheet s5A (Lys325, Glu327) and two residues on β-sheet C (Ser198, Arg186). Additionally, R10-4 exhibited its highest interaction density within the VNBD, accounting for ~61% of its total contacts. Among these, R10-4 formed several contacts (three each) with Thr120, Gln123, Lys141, and Lys145, all involved in direct binding with vitronectin except for Lys141 (Fig. 6A) (24–27). While ~25% of the residues interacting with R10-4 also engage variants T3–T5 within the VNBD, R10-4 displays a more expansive interaction profile that extends across multiple structural regions not observed in T3–T5 (Fig. 6B).

The truncated variant T2 exhibits the most distinct interaction pattern among the aptamer variants. Its contacts are concentrated on the posterior surface of PAI–1, primarily within the flexible joint region and adjacent α–helices (Fig. 6A). T2 engages several residues outside defined structural domains, Arg30, Val157, Gln159, and Arg300, and interacts with only one residue in the VNBD, Arg133 (Fig. 6). Within β–sheet C, T2 contacts Arg186, Ser195, and Ser198, the latter two being its only shared interactions with full–length R10–4. A relatively sparse interaction density is observed in T2, forming two contacts with Arg30, Val157, Arg287, and Arg 288. While all remaining interactions are engaged through a single contact (Fig. 6B). This distribution highlights a distinctive feature of T2: its regional significance is less concentrated than that of R10-4, suggesting a unique mechanism by which T2 modulates PAI-1 activity.

Truncated variants T3–T5 displayed more limited interaction profiles compared to R10-4. Nonetheless, they shared several interacting residues within the VNBD, particularly at Thr120, Gln123, and Lys145 (Fig. 6A). However, they did not interact extensively with PAI-1 residues in other structural domains. Occasional contacts with β-sheet A were observed, but none of these variants interacted with the RCL. These findings suggest that while the interactions within the VNBD may contribute to partial inhibitory activity retained by T3–T5, the full inhibitory potential of R10-4 likely depends on its broader interaction profile, particularly in the β-sheet s5A and the RCL. The localized contacts within two key regions—(i) VNBD and (ii) s5A and RCL—may play a critical role in anchoring the aptamer, stabilizing flexible domains, and modulating PAI-1 function.

### The reactive center loop engagement is essential for R10-4-mediated inhibition

**Figure 7.**
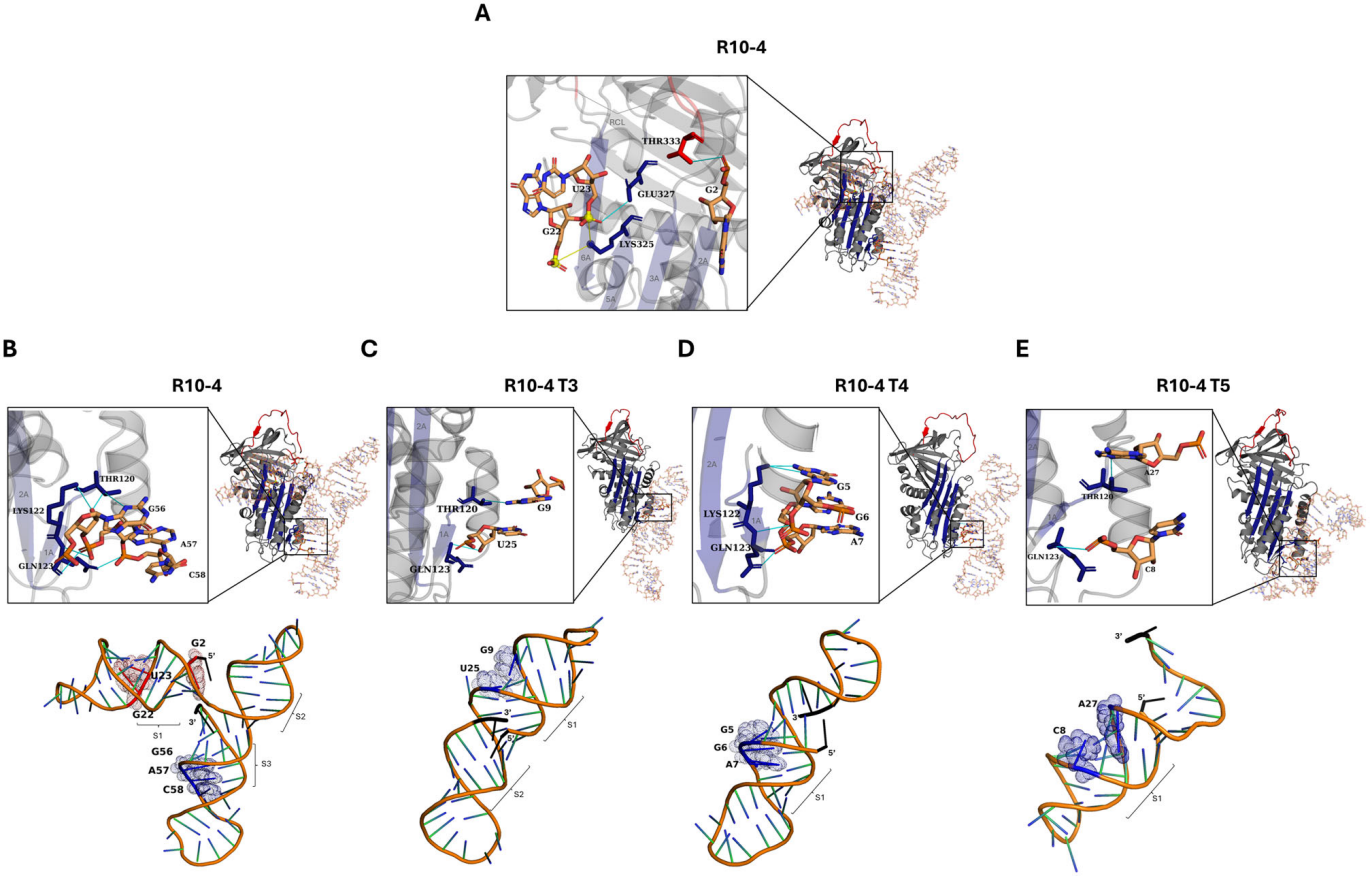
Key molecular interactions between PAI-1 and R10-4 full-length aptamer, and predicted functional motifs within R10-4, and its truncations. Protein and RNA residues involved in binding are shown in stick representation. Hydrogen bonds are depicted as cyan lines, salt bridges as yellow lines, and spheres indicate charge centers. Interacting PAI-1 residues located on the RCL are colored red, while those on β-sheet A are shown in blue. Aptamer residues are rendered in orange, with red and blue regions highlighting oxygen and nitrogen atoms, respectively. (**A**) Detailed view of key molecular interactions between active PAI-1 (PDB ID: 1B3K) and the R10-4 aptamer within the RCL/s5A region. PAI-1 residues in this region include THR333 (RCL), and GLU327 and LYS325 (s5A). (**B**–**E**) Detailed view of key interactions between PAI-1 and R10-4 aptamers and variants T3–T5 within the VNBD on the s1A strand (top panel). Predicted functional motifs within the R10-4 aptamer and its truncations, visualized in cartoon and sphere format. Nucleotides implicated in protein interaction with s5A regions of PAI-1 are labeled and overlaid with blue mesh spheres, and nucleotides implicated with RCL of PAI-1 are represented in red mesh spheres to denote regions of functional importance, as identified through structural modeling and PLIP-based interaction analysis (bottom panel). Visualization was performed in PyMOL.

To interrogate how residue-level engagement of R10-4 mediates PAI-1 inhibition, we examined the binding orientation and noncovalent interactions between PAI-1 and each aptamer variant. As shown earlier, truncating R10-4 abolished interactions with both s5A and the RCL, correlating with stem deletions that reorient the aptamer on the PAI-1 surface (Fig. 7A). This suggests that the three-branched architecture of R10-4 may be essential for anchoring the complex and enabling engagement with functionally critical regions (Fig. 7B).

Within the RCL, residue G2 of R10-4 forms two hydrogen bonds with Thr333, with Thr333 acting as both donor and acceptor. This dual interaction is strong and well-aligned spatially, positioning it within the hinge region of the RCL (Fig. 6A). In the s5A region, nucleotides U23 and G22 of R10-4 form salt bridges with Lys325. The interaction with U23 is particularly strong, driven by direct electrostatic interaction between the positively charged amine Lys325 and the negatively charged phosphate on U23. The interaction between G22 and Lys325 forms a longer hydrogen bond, which may contribute to long-range stabilization (Fig. 7A). Additionally, the interaction between U23 and Glu327 further refines binding specificity due to its proximity to the RCL insertion site and location on s5A (Fig. 7A and Supplementary Fig. S3). Collectively, these interactions may result in a steric hindrance that can delay conformational transition and obstruct the translocation of tPA following cleavage, thereby contributing to the inhibitory mechanism. The strength and multiplicity of these interactions imply that R10-4 not only stabilizes its binding orientation but also physically impedes conformational transitions required for PAI-1 activity.

Several studies have identified Lys325 as a critical residue in the latency transition of PAI-1, where it contributes to stabilization of the central β-sheet and facilitates RCL insertion [28–30]. Additionally, mutational analyses have shown that disruption of Lys325 does not impact protease inhibition for uPA reactions, indicating a tPA-specific role for this residue [29]. Glu327 is structurally adjacent to Lys325, suggesting a plausible functional relevance. R10-4’s engagement with both Lys325 and Glu327 supports its potential role in destabilizing the central β-sheet network, thereby impeding latency transition and rendering PAI-1 more susceptible to tPA cleavage. Aligning with our previous study, we have shown that R10-4 may promote latency. This disruption favors progression toward a substrate-like conformation, supporting the observed shift in PAI-1 behavior upon aptamer binding [10].

Significantly, the R10-4 forms two strong contacts at the base of the RCL loop, residue Thr333. A previous study demonstrated that mutation of Thr333 to Arg converts PAI-1 into a substrate, abolishing complex formation with plasminogen activators and resulting in complete cleavage, thereby implicating Thr333 as a critical determinant of inhibitory function [31]. Additionally, the humanized antibody CLB-10C12, which inhibits PAI-1’s interaction with tPA/uPA by directly blocking the RCL insertion, also interacts with Thr333 but not Glu327 [32, 33]. R10-4 interacts directly with Thr333, which reiterates the functional consequence of this mutation by destabilizing the RCL or altering its mobility, making it susceptible to protease cleavage. It is also possible that this interaction impairs complex formation via steric hindrance. When considered alongside R10-4’s additional contacts with Lys325 and Glu327, these interactions appear to cooperatively shift PAI-1 toward a substrate-like conformation in a tPA-specific manner. This is further supported by the observation that the truncated variants T3–T5, which lack interactions with these residues, do not impair the interaction of PAI-1 and tPA, implying that these residues are critical for R10-4′s inhibitory activity.

### VNBD interaction supports R10-4 inhibition, but full activity requires dual engagement with the reactive center loop

A unique feature of PAI-1 is its interaction with vitronectin, a multifunctional glycoprotein found in plasma and the extracellular matrix. Binding to vitronectin stabilizes the active conformation of PAI-1, extending its half-life and enhancing its inhibitory potency [27]. This interaction also influences cell adhesion, migration, and signaling, linking PAI-1 to broader processes such as angiogenesis and tumor progression [27]. We recently showed that R10-4 inhibits the migration of triple-negative breast cancer cells and inhibit secretion of cytokines important in angiogenesis [11]. Thus, understanding the molecular mechanisms of PAI-1 inhibition and its regulation by vitronectin is critical for deciphering the R10-4 inhibitory mechanism.

R10-4 engages the VNBD through a triad of residues—Thr120, Lys122, and Gln123—forming an extensive hydrogen-bonding network that anchors the aptamer within the flexible joint region of PAI-1 (Fig. 7B). G56 of R10-4 forms three hydrogen bonds with Thr120 and one with Lys122, including a strong electrostatic interaction between Lys122’s amine and the keto oxygen of G56. Gln123 forms three additional contacts, two of which involve G57, adopting a favorable donor geometry (Fig. 7B). Together, these interactions position R10-4 to engage all three residues within the VNBD triad fully.

This same triad of residues is a known target of the small-molecule PAI-1 inhibitor, TM5484, which binds the flexible joint region of PAI-1 and restricts its conformational mobility. In addition to its engagement with the VNBD triad, TM5484 interacts with residues located within adjacent α-heclices, hE and hF. Together, these interactions were shown to impair PAI-1’s inhibitory function by restricting RCL insertion and promoting substrate behavior via allosteric modulation within the flexible joint region [24]. Another small-molecule inhibitor, PAI-039, also binds within this region, making contacts with Arg118, Thr120, and Lys122. PAI-039 also interacts within a pocket formed by α-helix hE, β-sheet A (s1A), and α-helix F. These interactions ultimately impair PAI-1 activity by promoting its conversion to the latent form [34]. Another PAI-1 inhibitor that exploits this region is the monoclonal antibody MA42A2. MA42A2 similarly interacts with Lys122 and Glu123, but not with Thr120 [32, 33]. Although these inhibitors differ in their surrounding contacts, all converge on the VNBD triad, underscoring its functional importance [24, 34].

R10-4 reinforces that engagement of the triad within the VNBD can modulate PAI-1 conformational mobility. Building on this, variants T2–T5 also engage with this triad; however, they only interact with two of the three triad residues. Variants T2 and T3 form single hydrogen bonds with Thr120 and Gln123, while T5 forms one bond each with Lys122 and Gln123 (Fig. 7C–E). The lack of full engagement with the triad likely underlies their reduced inhibitory capacity, as contact with all three residues appears necessary to stabilize an allosteric pocket induced by R10-4.

Finally, while TM5484 and PAI-039 both disrupt the PAI-1/PA complex, increasing cleaved PAI-1—similar to R10-4—neither interacts with Lys325, Glu327, or Thr333 [8, 10, 24, 34]. This distinction suggests that R10-4 not only exploits an established VNBD regulatory mechanism but also modulates PAI-1 through an additional, previously unrecognized mechanism.

### R10-4 T2 variant exhibits distinct posterior PAI-1 interactions driving a unique bivalency mechanism of inhibition

**Figure 8.**
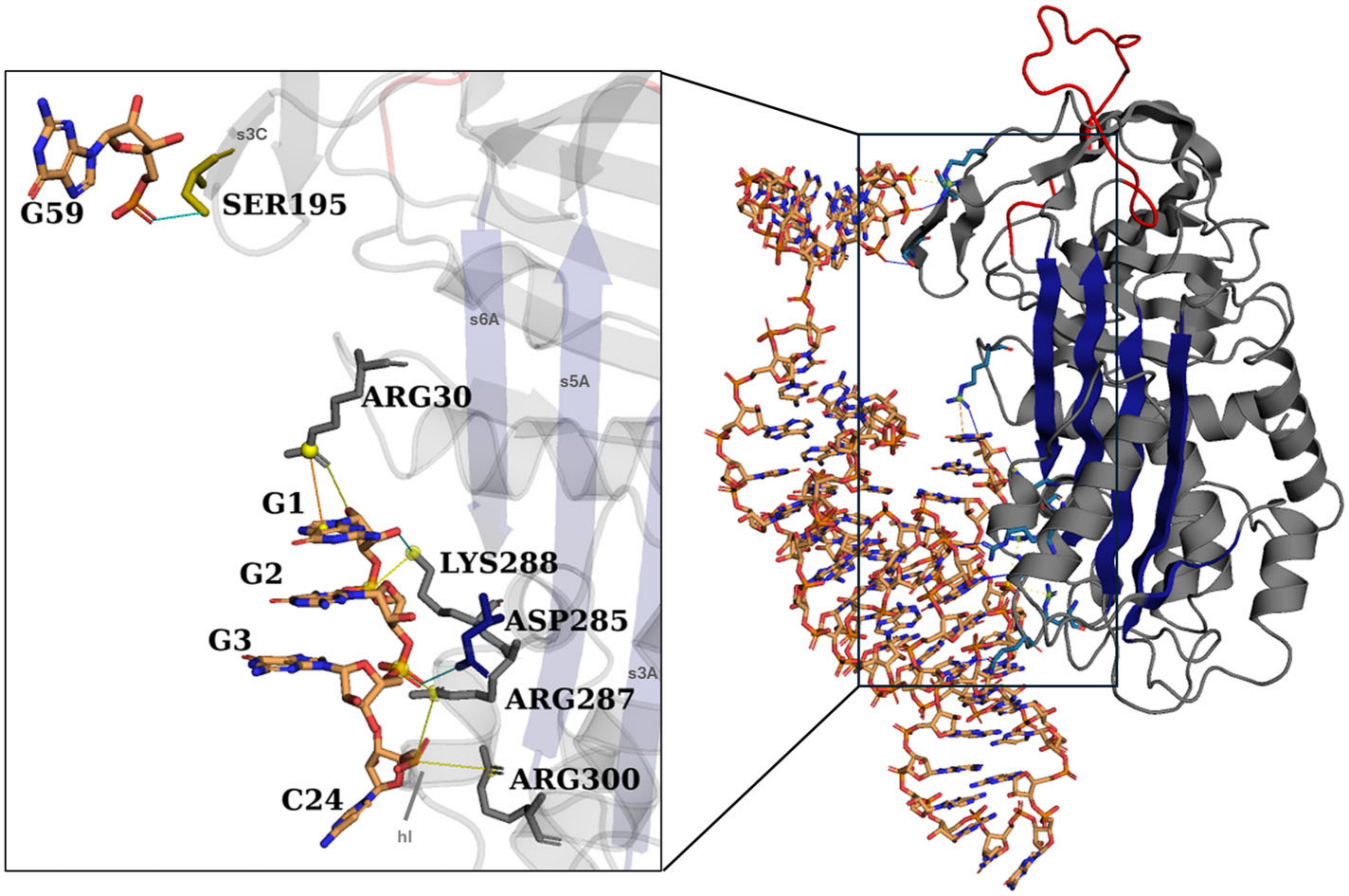
Core molecular interactions between the R10-4 T2 aptamer variant and active PAI-1 (PDB ID: 1B3K). The T2 aptamers is shown in stick representation and PAI-1 in cartoon. Interactions within the posterior flexible joint region are highlighted. Key RNA and protein residues involved in binding are displayed as sticks. Hydrogen bonds are shown as cyan lines, salt bridges as yellow lines, and π-cation interactions as orange lines; spheres mark charge centers. PAI-1 residues are colored by structural context: gray for helical or non-structured regions, blue for β-sheet A, and yellow for β-sheet C. R10-4 T2 residues are colored orange, with oxygen and nitrogen atoms indicated in red and blue, respectively. Visualization was performed in PyMOL.

We demonstrate that the R10-4 T2 variant retains its inhibitory activity through a distinct interaction profile on the posterior side of the PAI-1 interface. T2 forms contacts with Asp285, Arg287, Lys288, and Arg300. This network is considered strong, as T2 nucleotides G1–G3 and C24 form extensive salt bridge contacts with all indicated residues on the posterior flexible joint region (Fig. 8). This region overlaps with the direct binding interface to tPA, suggesting that this salt-bridge network between T2 and PAI-1 may compete with or disrupt tPA binding [35]. Additionally, several PAI-1-targeting antibodies, including MA-89D4 and Nb64, bind this region of PAI-1. Both antibodies disrupt PAI-1 function by binding epitopes centered on Arg300, Gln303, and Asp305, with Nb64 also engaging Asp297, thereby interfering with the final locking step of PAI-1/tPA complex formation [36–38]. The T2 R10-4 variant recapitulates the critical Arg300 engagement observed in these antibodies, while additional contacts with Lys288, Arg287, and Asp285 represent novel interactions not previously reported by known epitopes. The proximity and electrostatic complementarity of these residues suggest that T2 stabilizes a local conformation of PAI-1 that impedes the conformational transitions required for PAI-1/tPA locking, ultimately favoring substrate behavior.

A second inhibitory mechanism may involve delaying gate displacement. The Arg30–Asp193 electrostatic interaction is essential for this step in the latency transition [39]. T2 forms a hydrogen bond and π–cation interaction with Arg30, as well as a hydrogen bond with Ser195, potentially perturbing the Arg30–Asp193 pairing (Fig. 8). Such disruption could hinder gate movement, stall RCL translocation, and maintain PAI–1 in a conformation susceptible to tPA-mediated proteolytic cleavage.

## Conclusion

PAI–1 functions through the canonical serpin suicide–substrate mechanism. Upon interaction with its cognate protease, PAI-1 undergoes a substantial conformational rearrangement, forming a covalent serpin–protease complex that irreversibly inactivates the enzyme. Central to this process is the RCL, which behaves as a pseudosubstrate. Following cleavage at the P1–P1′ bond, the RCL inserts into β-sheet A of the serpin scaffold, thereby translocating the protease and distorting its active site. If RCL cleavage occurs without successful translocation, the protease is released, as observed with several small–molecule inhibitors [40, 41].

**Figure 9.**
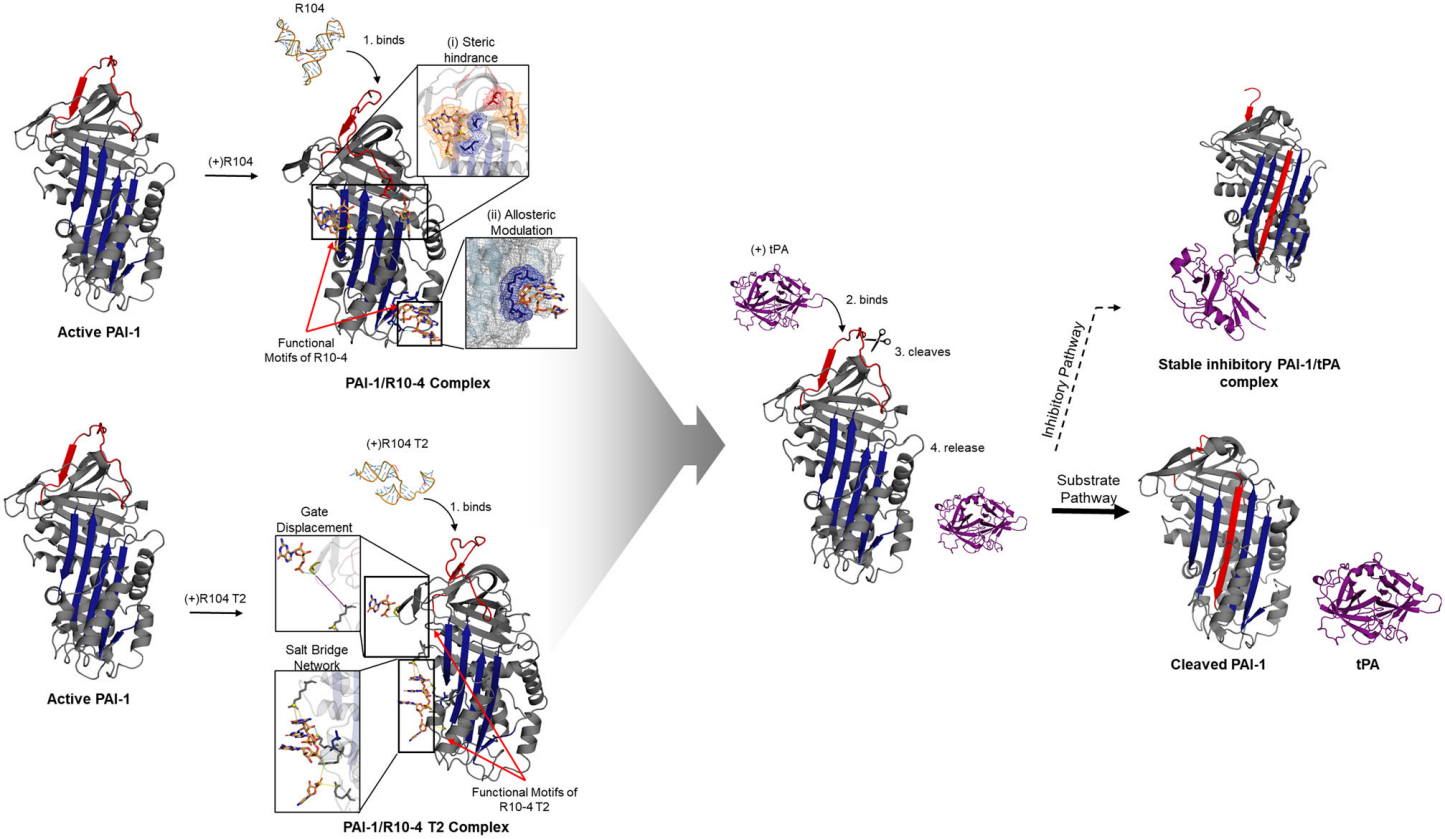
Theoretical model of PAI-1 modulation by R10-4 and R10-4 T2. The diagram depicts the sequential molecular events and structural transitions of active PAI-1 (PDB ID: 1B3K) upon initial interaction with R10-4 (top) and R10-4 T2 (bottom), then tPA. When active PAI-1 is pre-bound by R10-4, the aptamer forms a PAI-1:R10-4 complex that induces steric hindrance at the RCL/s5A region and allosteric modulation within the VNBD. When active PAI-1 is pre-bound by R10-4 T2, the aptamer forms a PAI-1:R10-4 T2 complex that interferes with gate displacements and constructs a salt bridge network within the posterior flexible joint region. Upon addition of tPA to these complexes, tPA binds, cleaves PAI-1, and is subsequently released, shifting PAI-1 away from the stable inhibitory PAI-1/tPA complex (PDB ID: 1EZX) and toward the substrate pathway and yielding cleaved PAI-1 (PDB ID: 3EOX) and free tPA (Bottom). Visualization was performed in PyMOL.

Data from this study reveal a previously uncharacterized ensemble of interactions that collectively suppresses PAI–1 activity. Although some contacts overlap with known inhibitory epitopes, the integrated interaction network identified here constitutes a distinct inhibitory signature with functional relevance for PAI–1 regulation. The full–length aptamer R10–4 exhibits a bivalent mode of action: (i) steric obstruction of the RCL–s5A interface, thereby impeding the latency transition, and (ii) restriction of conformational dynamics through engagement of a triad within the VNBD via allosteric modulation. Collectively, these interactions directly impede RCL insertion at the insertion site, leading to tPA binding, proteolytic cleavage, and tPA release. This ultimately results in a shift toward the substrate pathway with cleaved PAI-1 and free tPA as products (Fig. 8). The T2 variant also employs a novel dual mechanism: (i) delaying gate displacement and (ii) directly disrupting stable PAI-1/tPA complex formation by competing within the posterior flexible joint region through its salt-bridge network. This similarly promotes the substrate pathway as tPA cleaves and is released. (Fig. 9).

Collectively, these findings delineate critical PAI–1 residues targeted by R10–4 and R10–4 T2 and illustrate how aptamer–based inhibitors can exploit multiple structural domains to achieve potent and multifaceted inhibition.

## Supplementary Material

ugag026_Supplemental_File

## Data Availability

All data supporting the findings of this study are available within the article and its Supplementary Information files. Any additional data are available from the corresponding author upon reasonable request.
