# Supplementary material for: Aptamer-mediated modulation of PAI-1 function reveals a novel mechanism for restoring fibrinolytic balance
Source: NAR Mol Med. 2026 May 20;3(2):ugag026. doi: 10.1093/narmme/ugag026 (PMC13227106; doi:10.1093/narmme/ugag026)
Supplement: ugag026_Supplemental_File [file ugag026_supplemental_file.pdf]

SUPPLEMENTARY MATERIALS

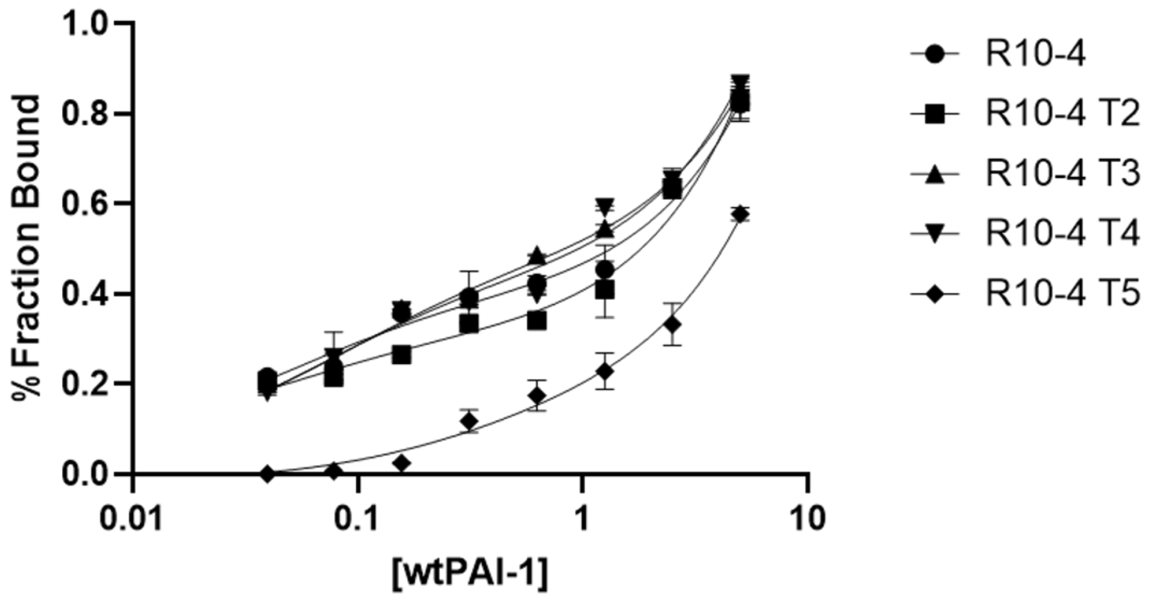

Supplementary Figure 1. SPR analysis of R10-4 and truncated variants, T2-T5 bound to PAI-1.

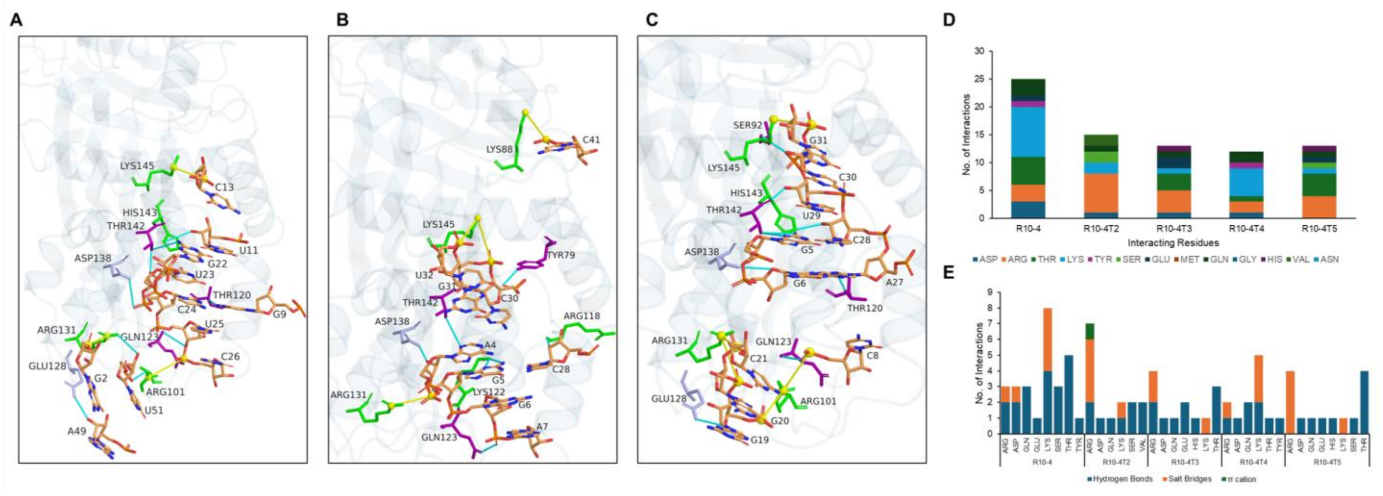

Supplementary Figure 2. Comprehensive interaction profile and residue-level interactions between R10-4 T3-T5 and active PAI-1 (PDB ID: 1B3K). (A) R10-4 T3 (B) R10-4 T4 (C) R10-4 T5. Across panels A-C, PAI-1 residues are color-coded by chemical properties: positively charged residues (Lys, Arg, His) in green; negatively charged residues (Asp, Glu) in light blue; and polar residues (Gln, Thr, Ser, Tyr) in purple. Interaction types are indicated as follows: cyan lines denote hydrogen bonds and yellow lines denote salt bridges. Spheres mark the calculated charge centers of each interacting residue in salt bridges. RNA aptamer atoms are shown in orange, with oxygen and nitrogen atoms highlighted in red and blue, respectively. (D) Stacked bar chart displaying the total number of interactions contributed by individual residues in all five aptamers. Each stack is represented by residues' identity with colors corresponding to specific amino acids (see legend). (E) Stacked bar chart displaying interaction types, including hydrogen bonds (blue), salt-bridges (orange), and π-cation interactions (green).

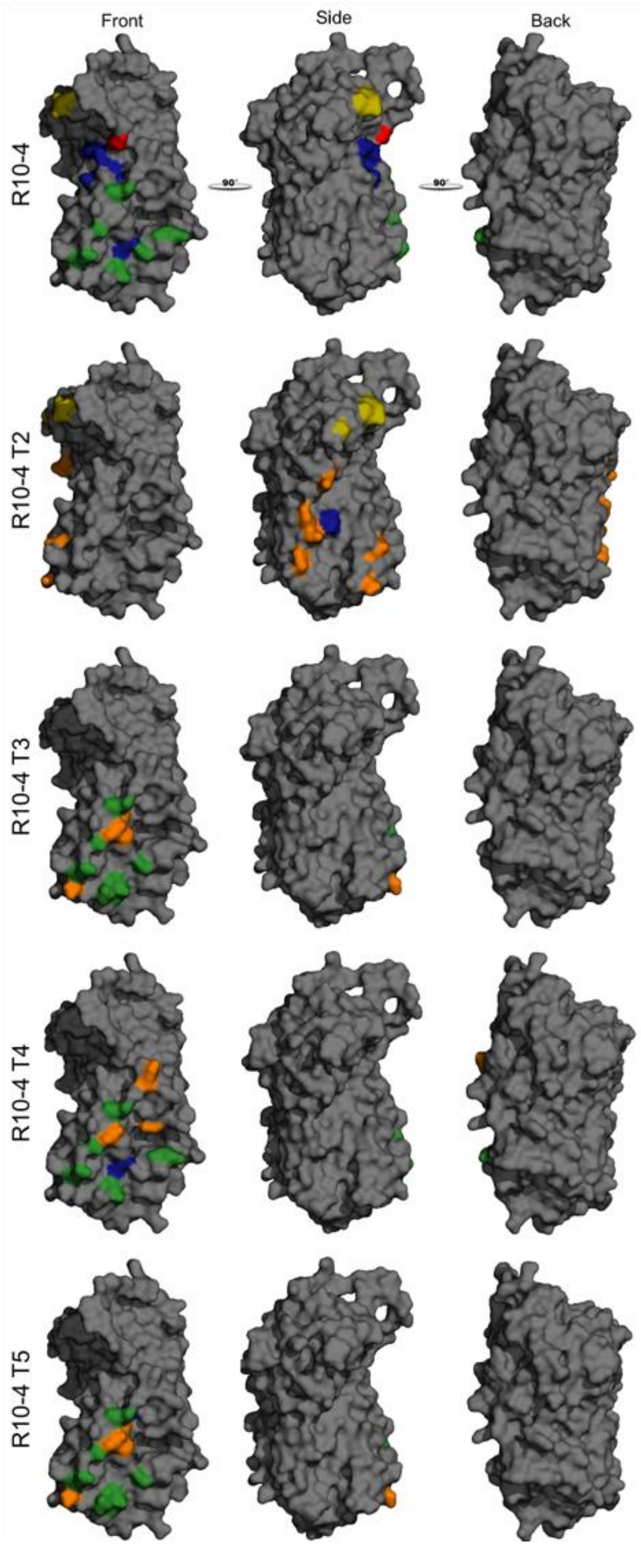

Supplementary Figure 3. Front, side, and back views of PAI-1 interactions with R10-4 and truncated variants (T2-T5).
